# Is the determination of C‐reactive protein really necessary in the diagnosis of Alpha‐1 antitrypsin deficiency?

**DOI:** 10.1111/crj.13494

**Published:** 2022-05-17

**Authors:** José María Hernández‐Pérez, Claudia Viviana López‐Charry

**Affiliations:** ^1^ Department of Neumology Hospital Universitario Nuestra Señora de Candelaria Santa Cruz de Tenerife Spain

Dear, Editor:

Alpha‐1 antitrypsin (AAT) is a medium‐sized, water‐soluble glycoprotein weighing 52 kDA with a half‐life of approximately 5 days and synthesized primarily by hepatocytes. Blood concentrations vary from 1 to 2 g/L (90–220 mg/dl), and as it is an acute phase reactant, its plasma levels can increase 2–4 times in response to inflammatory, infectious, or neoplastic stimuli.^1^

There are several studies that have evaluated and measured plasma concentrations of AAT according to the different AAT genotypes,^2^, ^3^, ^4^ but it was not until the study by Ferrarotti et al.^5^ that it was considered that as the first step in the algorithm diagnosis of AAT deficiency (AATD), C‐reactive protein (CRP) should be previously evaluated as a marker of the existence of conditions that could alter the levels of AAT in blood, since we could find very different values for the same genotype and for both make sure that we were in situations of apparent stability. In addition, it was mentioned that in the studies by Donato and Bomhorst, the range of the concentration of AAT levels for each genotype compared with those found in their study showed important differences, blaming the fact that in both studies, the levels of CRP were not previously measured and, therefore, they were extracted in situations of instability that altered these values.

For all these reasons, it was decided to carry out a prospective observational analytic study consecutively on 1510 subjects who attended the Pulmonology outpatient clinic for any reason, obtaining blood tests and performing genotyped for AAT in the same act, not including measurement of CRP values, most of the subjects were stable, but a percentage presented exacerbations (over 20%) and some of which led to hospital admissions. The study was conducted in accordance with the Declaration of Helsinki and approved by the corresponding ethics committee. All patients were informed of the objectives of the study and signed an informed consent. The inclusion criteria were wanting to participate in the study and being able to take both tests simultaneously. Genotyping was based on the use of so‐called hybridization probes or HybProbes,^6^ and AAT levels were assessed by immunonephelometry. The general results of the patients included in the study showed a median age of 57.5 with a standard deviation of 19.35, and 43.9% of the subjects were women; 19.3% had an active smoking habit, and 35.12% were ex‐smokers. Regarding the weight assessed by the body mass index (kg/m^2^), the median was 28.7, with a standard deviation of 6.09, with a range from 14.9 to 53.6 kg/m^2^. The most prevalent pathologies were obstructive airway diseases (bronchial asthma with 32.2% followed by COPD with 26.5%).

AAT values were analyzed, presenting a median of 123.5 mg/dl with a standard deviation (SD) of 31.8. The ranges of AAT levels obtained corresponding to each genotype were analyzed (Table 1). The overall detection success of deficient variants in the study was 31.4% (Pi*ZZ: 0.5%, Pi*SZ: 1.7%, Pi*SS: 1.5%, Pi*MZ: 7.4%, Pi*MS: 18.4%, and rare variants: 1.4%). The results showed that the measurement of the AAT levels together with the performance of the genotyping in the same act avoided errors and made it unnecessary to obtain the CRP, since it did not matter if the AAT values were increased due to inflammation, infection, or for another reason, since when carrying out the joint genotype, the diagnosis was reached safely, quickly, and efficiently.^7^ In addition, unnecessary visits and inconveniences for patients (repeat extractions) were avoided. In addition, if we compare the values obtained in our study with those reached by the studies by Donato,^3^ Bomhorst,^4^ Vidal,^2^ and Ferrarotti's own,^5^ we reach the conclusion that the difference does exist very small, especially in the lower limits for each genotype, giving greater value if possible, to the results obtained.

**TABLE 1:** Reference ranges (95%) of AAT level (mg/dl) found in a clinical population for different genotypes of the SERPINA1 gene, compared with other authors

Genotype	Clinical population			Vidal et al.^2^	Donatoet al.^3^	Ferrarottiet al.^5^	Bornhorstet al.^4^
	Mean	SD	Range^a^				
Pi*MM	135.9	25.9	99–197	103–200	100–273	102–175	102–254
Pi*MS	90.3	12.4	81–179	100–180	84–225	84–147	86–218
Pi*MZ	85.1	11.8	66–110	66–120	61–156	65–106	62–151
Pi*SS	90.3	12.4	75–125^b^	70–105	49–181	73–106^b^	43–154
Pi*SZ	60.1	9.9	44–82^b^	45–80	42–108	49–66^b^	38–108
Pi*ZZ	18.9	10.6	5–36^b^	10–40	15–57	32^b^	29–52

^a^ For the construction of the interval, individuals known, through subsequent analysis, to be carriers of rare deficiency alleles have been eliminated.

^b^ Full range shown due to small number of patients.

In a second phase, the AAT levels obtained for each genotype were divided according to whether or not the patient had an exacerbation (Table 2), confirming that in a stable situation, prior measurement of CRP levels is not necessary.

**TABLE 2:** Reference ranges (95%) of AAT level (mg/dl) found in patients with and without exacerbations for different genotypes of the SERPINA1 gene

Genotype	With exacerbations (*n* = 298) Median, CI (95%)	Without exacerbations (*n* = 1212) Median, CI (95%)
Pi*MM	153.7 (107–211)	129.4 (96–178)
Pi*MS	134.89 (111–182)	86.7 (82–107)
Pi*MZ	104.4 (85–110)	82.3 (66–96)
Pi*SS	93 (80–125)	85 (75–96)
Pi*SZ	67 (56–82)	56 (44–66)
Pi*ZZ	22.8 (12.2–36)	16.1 (5–25)

Therefore, in a patient with chronic respiratory disease (chronic obstructive pulmonary disease or bronchial asthma) and in whom it is desired to screen for the presence of associated AATD, the measurement of CRP levels would be obviated, if it is performed in the same act the determination of AAT levels together with genotyping,

## CONFLICT OF INTEREST

The authors declare no conflicts of interest related directly or indirectly to the contents of the manuscript.

## Data Availability

The data that support the findings of this study are openly available in Clinical History at https://www3.gobiernodecanarias.org/sanidad/scs/hospitaldelapalma.
